# Quantifying gut wall metabolism: methodology matters

**DOI:** 10.1002/bdd.2062

**Published:** 2017-02-14

**Authors:** Oliver J.D. Hatley, Christopher R. Jones, Aleksandra Galetin, Amin Rostami‐Hodjegan

**Affiliations:** ^1^ Simcyp Ltd (A Certara Company)Blades Enterprise CentreSheffieldS2 4SUUK; ^2^ DMPKHeptaresWelwyn Garden CityAL7 3AXUK; ^3^ Centre for Applied Pharmacokinetic Research, Manchester Pharmacy SchoolUniversity of ManchesterManchesterM13 9PTUK

## Perspective

### Background

Oral administration continues to be the dominant route for the dosing of small molecules. Therefore having adequate oral bioavailability remains a key component for the success of drug candidates. Amongst various factors determining the overall bioavailability, the role of the intestinal metabolism is commonly overlooked 1. Intestinal microsomes are commercially available, analogous to hepatic microsomes that are an essential part of the early drug discovery DMPK (Drug Metabolism and Pharmacokinetics) assessment. This disregard of intestinal metabolism is therefore not due to a lack of available *in vitro* tools, but a caveat of several confounding factors: the historical low activities in intestinal metabolism assays, and the absence of definitive scaling approaches for reliable quantitative extrapolation of the data generated. These factors are closely linked to the difficulties of producing reproducible intestinal microsomes and complications associated with heterogeneity of the small intestine relative to the liver, which may all explain why *in vitro–in vivo* extrapolation (IVIVE) of intestinal metabolism has not reached the same level of characterization as that of the liver. In this context, the published intestinal microsome preparation methods reveal a vast array of preparation techniques. These methodologies affect both the quality of the *in vitro* microsomal matrix, as well as confidence in defining absolute quantification of the intestinal metabolism component using scaling factors and IVIVE.

### Variation in methodologies – isolation of intestinal microsomes

**Figure 1:**
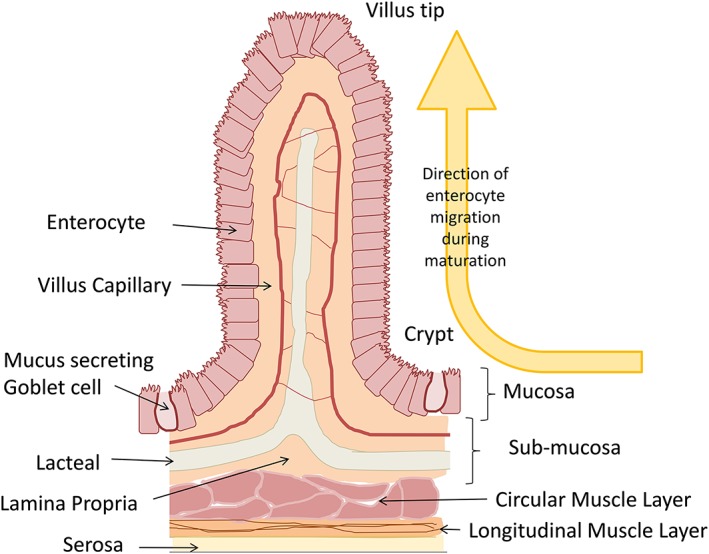
Generalized cross‐section of intestinal villus along the crypt to villus tip axis. The structure of the intestine includes the outer serosa, muscle and the sub‐mucosa and mucosa layers. The mucosa layer includes both enterocytes and mucus secreting goblet cells. During maturation the enterocytes migrate from the crypt to the villus tip before being sloughed off into the intestinal lumen. It should be noted that villus shape, width and number differs along the length of the intestine and between species 8

The low activity observed in intestinal microsomes has been linked to the method of intestinal microsomal preparation 2, 3. A traditional method for intestinal microsome preparation was scraping: the use of a glass slide or spatula to remove the mucosal layer of intestine before homogenization and preparation. The observed poor reproducibility, low abundances of cytochrome P450 (CYP), and high proportions of the degraded form of CYP (cytochrome P420 related to the spectrophotometric peak) indicated damage of CYP attributed to the ‘aggressive’ method of isolation, causing cell damage and exposure to proteolytic enzymes. The presence of these enzymes has been shown to be detrimental to the activity of prepared intestinal microsomes 2, 4, 5, 6; therefore, cocktails of protease inhibitors are an essential requirement for the preparation of intestinal microsomes 7. Contamination by a multitude of cell types in the mucosal layer of the intestine is an important additional factor that should not be overlooked (Figure 1). Further contamination by muscle and fat layers should also be considered when direct homogenization of intestine has been applied (e.g. 9, 10).

Mature enterocytes present near the outer surface of intestinal lumen at the tip of villi are the only cells with intrinsic metabolic potential 11, accounting for 25% of the total mucosal wet weight 12. In comparison, hepatocytes comprise >70% of liver cells and 80% of liver weight 13. Therefore, the isolation of a multitude of cell types in an intestinal preparation ultimately dilutes the sensitivity for identifying the metabolic potential of the isolate.

Enterocytes, however, compose up to 90% of the surface epithelium 8 (Figure 1). Consequently, a more selective approach is the use of chelating agents to facilitate enterocyte isolation using the elution method. This approach has been demonstrated to yield significantly higher intrinsic metabolic activity in rat and human intestinal tissues vs*.* scraped prepared microsomes 2, 3. Isolation of differing enterocyte layers reflecting the gradient of metabolic maturation of enterocytes as they migrate from the crypt to the villus tips has also been demonstrated using this technique 14, 15. However, despite the general consensus of adoption of this technique vs. scraping, the wide range of variations of preparation methodologies means that so far, no best practice for the preparation of intestinal microsomes has been established or critically assessed in the literature.

**Figure 2:**
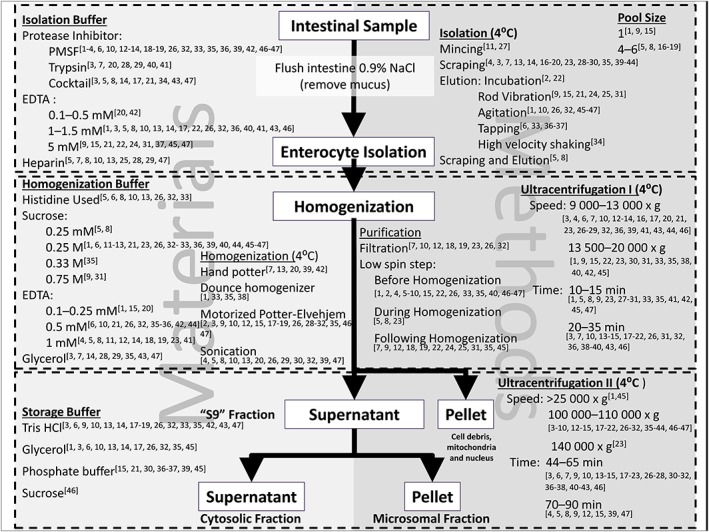
Schematic of published materials and preparation methods used for intestinal microsome preparation. References in Supplementary Material

Various sources are available in the literature that have utilized elution for the preparation of intestinal microsomes (Figure 2). However, the cumulative effects of differing procedures have so far not been assessed systematically. For example, intestinal sample length, enterocyte preparation method, homogenization procedures, protease inhibitors used, as well as buffer constituents vary among the studies. Even studies using the same elution agent (e.g. ethylenediaminetetraacetic acid (EDTA)), differ in the enterocyte preparation method. For example; vibration using metal rods 15; gentle agitation 14; tapping 16; or vigorously shaking 17 have been reported. Furthermore, studies vary in elution times and EDTA concentrations, and no systematic evaluation has taken place. Regional distributions of enzymes, as well as morphological changes to the structure vary along the length of the intestine 8, and therefore the impact of distributional changes mean study comparisons are often flawed, and also should be considered for its implications for IVIVE of intestinal first‐pass 11, 18.

Most recently, a methodology combining the initial scraping method, followed by isolation by elution was reported in the literature 7. The perceived benefit of this approach would be to allow for quicker and easier handling, since reduced preparation times were reported to minimize enzyme damage 4. Nevertheless, it must be considered that this approach yields loose agglomerated tissue, intestinal proteases, as well as mucus. As a result, final preparations may become contaminated, requiring addition of high protease inhibitor concentrations. Furthermore, the presence of mucus may impact on pellet formation, as reported previously 19. To overcome this, repeated ‘rinsing’ and low speed centrifugations have been employed in the initial isolation steps to help eliminate mucus and fat contaminants 14. Care should be taken when combining these steps with homogenization as this will liberate microsomal protein, which should therefore not be discarded, unlike as reported by Bruyere *et al*. 7.

Sonication is generally used in addition to rotor driven homogenization using a Potter‐Elvehjem tissue grinder 7, 10, based on the findings of Lindeskog *et al*. 20. Since the process of microsomal isolation is an inefficient process, the release of maximal microsomal protein is important both in terms of yields and for determining accurate measures of intestinal scaling factors. However, since CYP enzymes are sensitive to the sonication process 21, the balancing of impact of sonication intensity should be considered.

In addition, conflicting reports exist for the addition of glycerol, which is routinely utilized in liver microsome preparation 22. Glycerol has been reported to infer up to 30% protection to CYP during homogenization 23; most recently, no beneficial effect has been reported 7.

### The relevance to in vitro–in vivo extrapolation

A recent broad assessment of >300 drugs studied in humans has indicated that for 30% of the compounds, the fraction escaping intestinal metabolism (*F*_G_) was less than 0.8, highlighting the importance of incorporating intestinal metabolism in both bioavailability and dose predictions in drug discovery and development 24. This may be of particular significance when considering drugs with an oral bioavailability lower than 30%, for which the understanding of a high degree of inter‐individual variability in exposure may be critical, particularly for drugs with a low therapeutic range 25. The long term stability and metabolic competence of microsomes are important characteristics of these *in vitro* tools. Quantitative IVIVE, within the physiologically based paradigm, requires organ specific scaling factors that relate the activity observed in *in vitro* protein to the whole organ. These have been applied to extrapolate UDP‐glucuronosyltransferase (UGT) intrinsic clearance data 26. However, a lack of characterization of microsomal scaling factors for intestinal IVIVE and corresponding regional differences limits the robustness of quantitative IVIVE of intestinal metabolism from microsomes. Alternatively, extrapolation can be achieved by accounting for the abundance of relevant metabolic enzymes in the small intestine as reported in the case of CYP3A4 17, 27, 28. At present, emerging LC–MS/MS based protein expression data for other metabolic enzymes in the small intestine are still sparse. In addition, any uncertainties about the main enzymatic route of elimination favour the use of a generic intestinal microsomal scaling factor.

Since the process of microsomal isolation results in the loss of microsomal protein during preparation, corrections for losses should be applied to the scaling factor. Therefore, it is necessary to use a microsomal specific marker in order to measure the total content in the starting homogenate vs. the final microsomal fraction. Incorporation of the microsomal recovery is therefore an important element in determining reliable scaling factors for IVIVE and this approach has been well established and characterized for the liver 22, 29, 30. In contrast for the intestine, only a handful of studies have been reported for human 18 and dog tissue 31, 32 (Table 1), and therefore requires a focused effort. It should also be noted from Table 1 that meta‐analysis of intestinal scaling factors is compromised by the preparation methods, segment length and regions used, and pooling of different sexes.

**Table 1:** Literature reported intestinal microsomal protein IVIVE scaling factors

Scalar	Methodology	Rat 10, 15, 33, 34	Dog 31, 32	Human 9, 18
Microsomal protein per g intestine (MPPGI)	Direct homogenization	2.5^d^^,^ g ^,^ b	–	3.9^d^^,^ c
	Elution	7.8^d^^,^ e ^,^ b	13.8^a^	–
		2.3^d^	6.8^a^	
		9.7^b^		
	Scraping	10^d^^,^ g ^,^ b	–	3.1^a^
Total mg microsomal protein per intestine (MPI)	Direct homogenization	17^d^	–	3155^d^^,^ f ^,^ c
	Elution	54^d^^,^ e	4991	–
		16^d^^,^ g	2028	
		102.4^e^		
	Scraping	69^d^^,^ b	–	2978

Rat: Male Wistar *n* = 6 15, 34, *n* = 18 33. Unknown sex and strain for *n* = 4 10. Dog (Beagle): mixed sex donors, *n* = 4 in each study 31, 32. Human: eight mixed sex donors 9. Seven mixed sex donors 18.

a Regional weighted mean.

b Proximal intestine segment.

c Mixed regional samples.

d No correction for losses during preparation.

e Segment microsomal protein yield extrapolated from half to whole of intestine.

f Based on intestinal weight of 809 g 18.

g Based on intestinal weight of 6.9 g 36.

The most comprehensive assessment to date is for dog (beagle), where in addition to the shown weighted mean and sex‐pooled data, individual and regional scalars have been characterized. However from the limited data available, it should be noted that differences within the same general preparation technique shows a 2‐fold difference in scalars, although the potential for the impact of the different geographical locations of the donor colonies should also be considered. This again highlights the necessity for characterization of the study system in order to establish confidence in IVIVE strategies.

## Conclusion

The overall potential impact of multitude of factors critically discussed above on total CYP contents, resultant activity and intestinal scalars have not been a focus of studies to date. However, this is an important first step in the quantitative prediction of intestinal metabolism requiring systematic assessment. Given that the multiple techniques employed for enterocyte and microsomal preparation have the potential to influence the microsomal protein yield, the choice of method may affect the resulting scaling factors 35. Understanding this is a key requisite to future successful intestinal IVIVE. Therefore, in the absence of robust intestinal scaling strategies it is recommended that the system used is characterized. The impact of the above highlighted critical steps in intestinal microsome preparation, and an optimized methodology has been suggested in an accompanying manuscript 33.

## Conflict of Interest

The authors declare that there is no conflict of interest regarding the publication of this paper.

## Supporting information

Supporting info item
