# Supplementary material for: Quantifying gut wall metabolism: methodology matters
Source: Biopharm Drug Dispos. 2017 Feb 14;38(2):155–60. doi: 10.1002/bdd.2062 (PMC5412859; doi:10.1002/bdd.2062)
Supplement: Supplementary file 1 — Supporting info item [file BDD-38-155-s001.pdf]

Supplementary Material

References for Figure 1:

1. Cotreau, M.M., et al., Methodologies to study the induction of rat hepatic and intestinal cytochrome P450 3A at the mRNA, protein, and catalytic activity level. J Pharmacol Toxicol Methods, 2000. 43(1): p. 41-54.
2. Watkins, P.B., et al., Identification of glucocorticoid-inducible cytochromes P-450 in the intestinal mucosa of rats and man. J Clin Invest, 1987. 80(4): p. 1029-36.
3. Emoto, C., et al., Characterization of cytochrome P450 enzymes involved in drug oxidations in mouse intestinal microsomes. Xenobiotica, 2000. 30(10): p. 943-53.
4. Damre, A., S.R. Mallurwar, and D. Behera, Preparation and characterization of rodent intestinal microsomes: comparative assessment of two methods. Indian J Pharm Sci, 2009. 71(1): p. 75-7.
5. Bruyere, A., et al., Development of an optimized procedure for the preparation of rat intestinal microsomes: comparison of hepatic and intestinal microsomal cytochrome P450 enzyme activities in two rat strains. Xenobiotica, 2009. 39(1): p. 22-32.
6. Mohri, K. and Y. Uesawa, Enzymatic activities in the microsomes prepared from rat small intestinal epithelial cells by differential procedures. Pharm Res, 2001. 18(8): p. 1232-6.
7. Jones, D.P., R. Grafstrom, and S. Orrenius, Quantitation of hemoproteins in rat small intestinal mucosa with identification of mitochondrial cytochrome P-450. J Biol Chem, 1980. 255(6): p. 2883-90.
8. Bruyere, A., et al., Effect of Variations in the Amounts of P-Glycoprotein (ABCB1), BCRP (ABCG2) and CYP3A4 along the Human Small Intestine on PBPK Models for Predicting Intestinal First Pass. Mol Pharm.
9. Dawson, J.R. and J.W. Bridges, Intestinal microsomal drug metabolism: A comparison of rat and guinea-pig enzymes, and of rat crypt and villous tip cell enzymes Biochemical Pharmacology, 1981. 30(17): p. 2415-2420.
10. Zhang, Q.Y., et al., Characterization of human small intestinal cytochromes P-450. Drug Metab Dispos, 1999. 27(7): p. 804-9.
11. de Waziers, I., et al., Cytochrome P 450 isoenzymes, epoxide hydrolase and glutathione transferases in rat and human hepatic and extrahepatic tissues. J Pharmacol Exp Ther, 1990. 253(1): p. 387-94.
12. Paine, M.F., et al., Characterization of interintestinal and intraintestinal variations in human CYP3A-dependent metabolism. J Pharmacol Exp Ther, 1997. 283(3): p. 1552-62.
13. Powell, M., Kinetics of Cytochrome P450 Enzymes in the Canine Liver and Small Intestine, in School of Pharmacy and Pharmaceutical Sciences. 2006, University of Manchester.
14. Takemoto, K., et al., Catalytic activities of cytochrome P450 enzymes and UDP-glucuronosyltransferases involved in drug metabolism in rat everted sacs and intestinal microsomes. Xenobiotica, 2003. 33(1): p. 43-55.
15. Koster, A.S. and J. Noordhoek, Glucuronidation in the rat intestinal wall. Comparison of isolated mucosal cells, latent microsomes and activated microsomes. Biochem Pharmacol, 1983. 32(5): p. 895-900.
16. Prueksaritanont, T., et al., Comparative studies of drug-metabolizing enzymes in dog, monkey, and human small intestines, and in Caco-2 cells. Drug Metab Dispos, 1996. 24(6): p. 634-42.
17. Shiratani, H., et al., Species differences in UDP-glucuronosyltransferase activities in mice and rats. Drug Metab Dispos, 2008. 36(9): p. 1745-52.
18. Paine, M.F., et al., The human intestinal cytochrome P450 "pie". Drug Metab Dispos, 2006. 34(5): p. 880-6.
19. Mouly, S. and M.F. Paine, P-glycoprotein increases from proximal to distal regions of human small intestine. Pharm Res, 2003. 20(10): p. 1595-9.
20. Komura, H., et al., Species difference in nisoldipine oxidation activity in the small intestine. Drug Metab Pharmacokinet, 2002. 17(5): p. 427-36.

21. Bock, K.W., et al., Tissue-specific regulation of canine intestinal and hepatic phenol and morphine UDP-glucuronosyltransferases by beta-naphthoflavone in comparison with humans. Biochem Pharmacol, 2002. 63(9): p. 1683-90.
22. Weiser, M.M., Intestinal epithelial cell surface membrane glycoprotein synthesis. I. An indicator of cellular differentiation. J Biol Chem, 1973. 248(7): p. 2536-41.
23. Hubscher, G., G.R. West, and D.N. Brindley, Studies on the fractionation of mucosal homogenates from the small intestine. Biochem J, 1965. 97(3): p. 629-42.
24. Borm, P., A. Frankhuijzen-Sierevogel, and J. Noordhoek, Time and dose dependence of 3-methylcholanthrene-induced metabolism in rat intestinal mucosal cells and microsomes. Biochem Pharmacol, 1982. 31(22): p. 3707-10.
25. Borm, P.J., A. Frankhuijzen-Sierevogel, and J. Noordhoek, Kinetics of in vitro O-deethylation of phenacetin and 7-ethoxycoumarin by rat intestinal mucosal cells and microsomes. The effect of induction with 3-methylcholanthrene and inhibition with alpha-naphthoflavone. Biochem Pharmacol, 1983. 32(10): p. 1573-80.
26. Fasco, M.J., et al., Rat small intestinal cytochromes P450 probed by warfarin metabolism. Mol Pharmacol, 1993. 43(2): p. 226-33.
27. Pacifici, G.M., et al., Tissue distribution of drug-metabolizing enzymes in humans. Xenobiotica, 1988. 18(7): p. 849-56.
28. Stohs, S.J., et al., The isolation of rat intestinal microsomes with stable cytochrome P-450 and their metabolism of benzo(alpha) pyrene. Arch Biochem Biophys, 1976. 177(1): p. 105-16.
29. Lindeskog, P., et al., Isolation of rat intestinal microsomes: partial characterization of mucosal cytochrome P-450. Arch Biochem Biophys, 1986. 244(2): p. 492-501.
30. Hoensch, H., et al., Oxidative metabolism of foreign compounds in rat small intestine: cellular localization and dependence on dietary iron. Gastroenterology, 1976. 70(6): p. 1063-70.
31. Shirkey, R.S., J. Chakraborty, and J.W. Bridges, Comparison of the drug metabolising ability of rat intestinal mucosal microsomes with that of liver. Biochem Pharmacol, 1979. 28(18): p. 2835-9.
32. Zhang, Q.Y., et al., Characterization of rat small intestinal cytochrome P450 composition and inducibility. Drug Metab Dispos, 1996. 24(3): p. 322-8.
33. Bonkovsky, H.L., et al., Cytochrome P450 of small intestinal epithelial cells. Immunochemical characterization of the increase in cytochrome P450 caused by phenobarbital. Gastroenterology, 1985. 88(2): p. 458-67.
34. von Richter, O., et al., Cytochrome P450 3A4 and P-glycoprotein expression in human small intestinal enterocytes and hepatocytes: a comparative analysis in paired tissue specimens. Clin Pharmacol Ther, 2004. 75(3): p. 172-83.
35. Pinkus, L.M. and H.G. Windmueller, Phosphate-dependent glutaminase of small intestine: localization and role in intestinal glutamine metabolism. Arch Biochem Biophys, 1977. 182(2): p. 506-17.
36. Yoon, I.S., et al., Pharmacokinetics and first-pass elimination of metoprolol in rats: contribution of intestinal first-pass extraction to low bioavailability of metoprolol. Xenobiotica, 2011. 41(3): p. 243-51.
37. Lampen, A., et al., Metabolism and transport of the macrolide immunosuppressant sirolimus in the small intestine. J Pharmacol Exp Ther, 1998. 285(3): p. 1104-12.
38. Fitzsimmons, M.E. and J.M. Collins, Selective biotransformation of the human immunodeficiency virus protease inhibitor saquinavir by human small-intestinal cytochrome P4503A4: potential contribution to high first-pass metabolism. Drug Metab Dispos, 1997. 25(2): p. 256-66.
39. Lu, X., C. Li, and D. Fleisher, Cimetidine sulfoxidation in small intestinal microsomes. Drug Metab Dispos, 1998. 26(9): p. 940-2.
40. Keelan, M., et al., Dietary omega 3 fatty acids and cholesterol modify enterocyte microsomal membrane phospholipids, cholesterol content and phospholipid enzyme activities in diabetic rats. Lipids, 1994. 29(12): p. 851-8.
41. Eeckhoudt, S.L., Y. Horsmans, and R.K. Verbeeck, Differential induction of midazolam metabolism in the small intestine and liver by oral and intravenous dexamethasone pretreatment in rat. Xenobiotica, 2002. 32(11): p. 975-84.

42. Kanazu, T., et al., Assessment of the hepatic and intestinal first-pass metabolism of midazolam in a CYP3A drug-drug interaction model rats. Xenobiotica, 2005. 35(4): p. 305-17.
43. Kurosawa, S., et al., Effect of ursodeoxycholic acid on the pharmacokinetics of midazolam and CYP3A in the liver and intestine of rats. Xenobiotica, 2009. 39(2): p. 162-70.
44. Chhabra, R.S., R.J. Pohl, and J.R. Fouts, A comparative study of xenobiotic-metabolizing enzymes in liver and intestine of various animal species. Drug Metab Dispos, 1974. 2(5): p. 443-7.
45. Hirunpanich, V., K. Murakoso, and H. Sato, Inhibitory effect of docosahexaenoic acid (DHA) on the intestinal metabolism of midazolam: in vitro and in vivo studies in rats. Int J Pharm, 2008. 351(1-2): p. 133-43.
46. Heikkinen AT, Friedlein A, Lamerz J, Jakob P, Cutler P, Fowler S, et al. Mass spectrometry-based quantification of CYP enzymes to establish in vitro/in vivo scaling factors for intestinal and hepatic metabolism in beagle dog. Pharm Res. 2012 Jul;29(7):1832-42.
47. Heikkinen AT, Friedlein A, Matondo M, Hatley OJ, Petsalo A, Juvonen R, et al. Quantitative ADME Proteomics - CYP and UGT Enzymes in the Beagle Dog Liver and Intestine. Pharm Res. 2014 Jul 18.
